# Neuronal and behavioural modulations by pathway-selective optogenetic stimulation of the primate oculomotor system

**DOI:** 10.1038/ncomms9378

**Published:** 2015-09-21

**Authors:** Ken-ichi Inoue, Masahiko Takada, Masayuki Matsumoto

**Affiliations:** 1 Systems Neuroscience Section, Primate Research Institute, Kyoto University, Inuyama, Aichi 484-8506, Japan; 2 Division of Biomedical Science, Faculty of Medicine, University of Tsukuba, Tsukuba, Ibaraki 305-8577, Japan

## Abstract

Optogenetics enables temporally and spatially precise control of neuronal activity *in vivo*. One of the key advantages of optogenetics is that it can be used to control the activity of targeted neural pathways that connect specific brain regions. While such pathway-selective optogenetic control is a popular tool in rodents, attempts at modulating behaviour using pathway-selective optogenetics have not yet been successful in primates. Here we develop a methodology for pathway-selective optogenetics in macaque monkeys, focusing on the pathway from the frontal eye field (FEF) to the superior colliculus (SC), part of the complex oculomotor network. We find that the optogenetic stimulation of FEF projections to the SC modulates SC neuron activity and is sufficient to evoke saccadic eye movements towards the response field corresponding to the stimulation site. Thus, our results demonstrate the feasibility of using pathway-selective optogenetics to elucidate neural network function in primates.

## Abstract (web summary)

Pathway-selective optogenetics enables the precise control of neural activity in targeted pathways connecting specific brain regions. Here the authors provide the first demonstration that pathway-selective optogenetics can modulate neural activity and behaviour in non-human primates.

## Graphical abstract

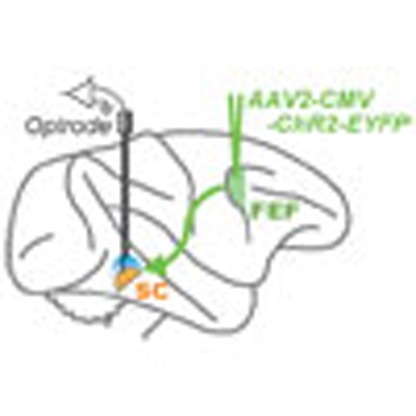

One of the major goals in systems neuroscience is to understand how the brain generates behaviour. To do so, it is critical not only to understand the structure and function of the individual brain regions that contribute to behaviour, but also to understand how these regions interact. Electrical microstimulation and pharmacological manipulation have long been used to investigate the causal contribution of individual brain regions to behaviour. However, these techniques lack the precision necessary to study functional contributions of specific cell types and their interactions with other brain regions. For example, electrical microstimulation or pharmacological activation of a particular brain region simultaneously activates all of the output pathways from that region, preventing an experimenter from studying the role of the specific output pathway.

Optogenetics has recently provided a powerful tool to address this issue^1^,^2^. Using genetic techniques, molecularly defined neurons can be genetically modified to express channelrhodopsin-2 (ChR2), a blue-light-sensitive cation channel. The photostimulation of ChR2 expressed on axon terminals in recipient brain structures induces a synaptic response and can therefore evoke signal transmission between two brain regions. Such pathway-selective optogenetics has been met with widespread success in modulating behaviour in rodents, and has advanced our understanding of the roles of particular neural pathways in a variety of behaviours^3^,^4^,^5^,^6^. To the best of our knowledge, however, there are currently no successful reports of behavioural modulations using pathway-selective optogenetics in nonhuman primates. Although there have been some successful attempts at using optogenetics, in general, to modulate behaviour in primates, these previous studies have used non-pathway-selective optogenetics, rendering them similar to electrical microstimulation and pharmacological manipulations^7^,^8^,^9^,^10^,^11^.

In the present work, we develop the methodology for pathway-selective optogenetics in macaque monkeys (*Macaca mulatta*). We focus on the oculomotor system, which is well developed in primates and consists of a complex network of cortical and subcortical regions including the frontal eye field (FEF), lateral intraparietal area and superior colliculus (SC)^12^,^13^,^14^,^15^,^16^. Among this network of regions, here we examine the neuronal and behavioural effects of optogenetically activating the pathway from the FEF to the SC (Fig. 1a). We find that our optogenetic approach induces not only neuronal but also behavioural modulations in macaque monkeys.

## Results

### ChR2 expression in the FEF and SC

**Figure 1:**
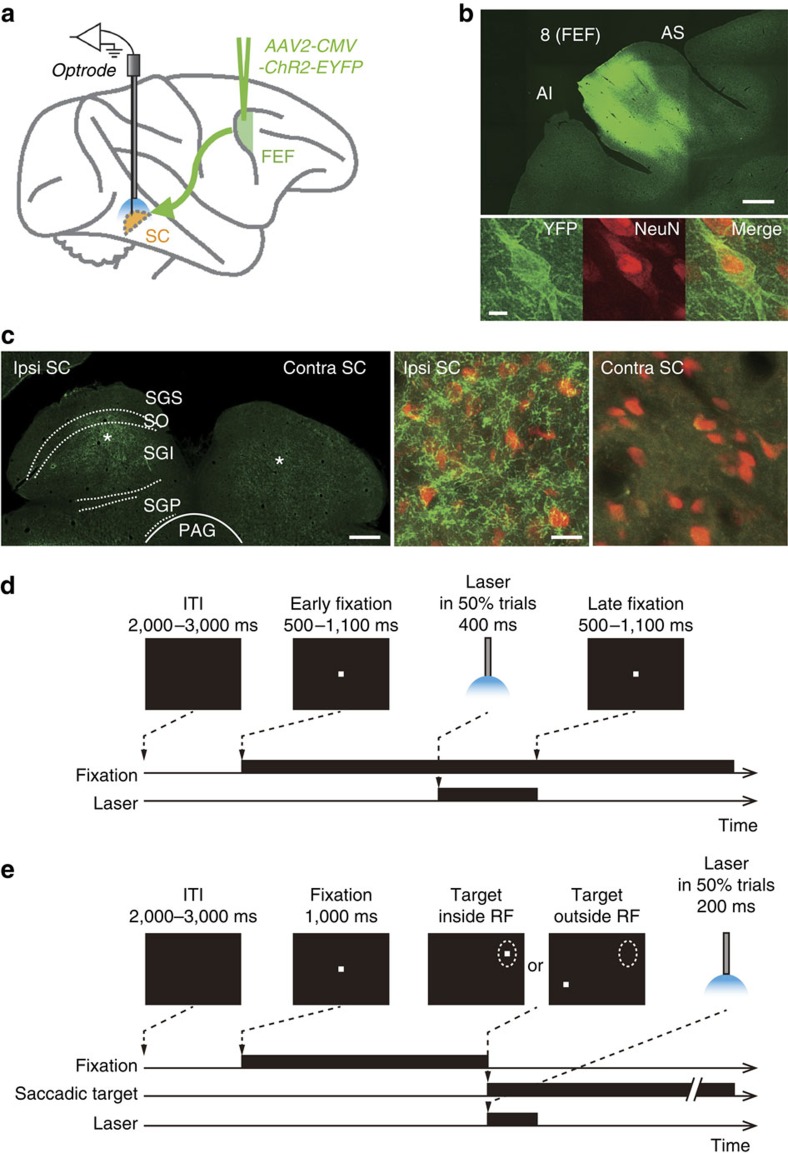
Experimental design and histological analysis. (**a**) Schema of experimental configuration. (**b**) ChR2-EYFP expression in the FEF. Top, wide-field fluorescent image of a coronal section through the FEF. Bottom, immunofluorescence of an FEF neuron example (left, YFP; middle, NeuN; right, YFP and NeuN). Scale bars, 2 mm for the top panel and 10 μm for the bottom panels. AI, inferior limb of the arcuate sulcus; AS, superior limb of the arcuate sulcus. (**c**) ChR2-EYFP expression in the SC. Left, wide-field immunofluorescent image of a coronal section through the SC. Middle and right, immunofluorescence of YFP-expressing axons in the ipsilateral and contralateral SC, respectively. NeuN-expressing SC neurons are shown in red. Asterisks in the left panel indicate the locations of the ipsilateral and contralateral images in the SC. Scale bars, 1 mm for the left panel and 20 μm for the middle and right panels. PAG, periaqueductal grey; SGI, stratum griseum intermediale; SGP, stratum griseum profundum; SGS, stratum griseum superficiale; SO, stratum opticum. (**d**) Fixation task. (**e**) Visually guided saccade task.

To deliver the ChR2(H134R) gene into targeted neurons, we injected an adeno-associated virus type 2 vector (AAV2-CMV-ChR2-EYFP) into the FEF of one hemisphere in two monkeys (monkeys LX and GN). At the end of the experiments in monkey LX, we histologically confirmed the expression of the ChR2-EYFP chimeric protein. Many ChR2-positive neurons were found in the FEF (Fig. 1b). We also observed many ChR2-positive axon terminals in the intermediate layer of the SC ipsilateral to the vector-injected hemisphere (Fig. 1c). ChR2-positive terminals were much less dense in the contralateral SC, consistent with an anatomical observation that the FEF sends only weak projections to the contralateral SC^17^. These data indicated that ChR2 was successfully expressed on the axon terminals of FEF neurons within the SC.

### Neuronal modulation evoked by optical stimulation

We first examined whether optical stimulation of FEF axon terminals affected postsynaptic single-neuron firing in the SC. Using optrodes (an optic fibre attached to a recording electrode), we stimulated the SC using 473-nm blue laser light and simultaneously recorded single-unit activity (monkey LX, *n*=35; monkey GN, *n*=46 neurons) while the monkey was performing a visual fixation task (Fig. 1d). During the task, the monkey was required to fixate a central point. On half of the trials the SC was stimulated with the blue light, while on the other half the light was absent.

**Figure 2:**
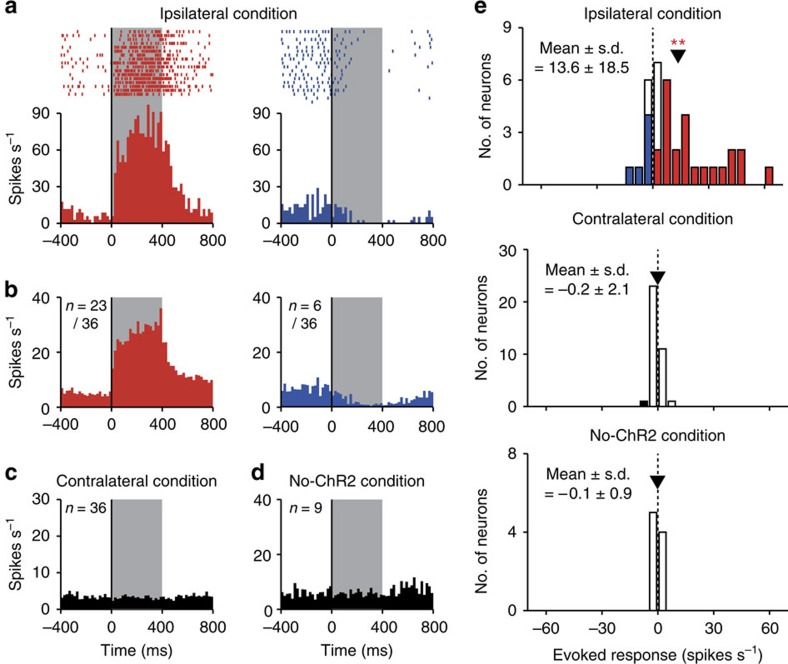
Neuronal modulations by the optical stimulation in the fixation task. (**a**) Activity of two SC neuron examples that were excited (left) and suppressed (right) in the ipsilateral condition. Histograms (20 ms bins) and rasters are aligned at the onset of the optical stimulation. Grey areas indicate the period of laser light emission. (**b**) Averaged activities of the excited (*n*=23, left) and suppressed neurons (*n*=6, right) in the ipsilateral condition. (**c**,**d**) Averaged activities in the contralateral (*n*=36 (**c**)) and no-ChR2 conditions (*n*=9 (**d**)). (**e**) Distribution of the evoked-response magnitude of each SC neuron in the ipsilateral (*n*=36, top), contralateral (*n*=36, middle) and no-ChR2 conditions (*n*=9, bottom). Filled bars indicate neurons showing a significant excitation or suppression (Wilcoxon signed-rank test, *P*<0.05). Arrowheads indicate the mean response magnitudes. Double asterisks indicate a significant deviation from 0 (Wilcoxon signed-rank test, *P*<0.01).

Through the optical stimulation of FEF axon terminals in the ipsilateral SC (ipsilateral condition), we found that 23 of 36 recorded neurons exhibited an excitatory response during laser light emission and 6 of 36 neurons exhibited significant suppression (Wilcoxon signed-rank test, *P*<0.05) (see Fig. 2a for excited and suppressed neuron examples, and Fig. 2e top for the evoked-response magnitude of each neuron). The averaged activity of the excited or suppressed neurons exhibited a clear sustained peak or trough, respectively, during laser light emission (Fig. 2b). We also assessed the effects of optically stimulating the contralateral SC (contralateral condition), where FEF axon terminals expressed little ChR2. Of 36 recorded neurons, only one showed a significant modulation (Wilcoxon signed-rank test, *P*<0.05; Fig. 2e middle) that might be due to weaker projections from the contralateral FEF^17^. The averaged activity did not show any clear modulation (Fig. 2c). As a further control, we tested the effect of the laser light on SC neuron activity before the vector injection in monkey GN (no-ChR2 condition). None of nine recorded neurons exhibited a significant modulation (Wilcoxon signed-rank test, *P*>0.05; Fig. 2d,e bottom). Thus, the laser light itself did not modulate SC neuron activity. The proportion of neurons with a significant modulation was significantly higher in the ipsilateral condition than in the contralateral and no-ChR2 conditions (*χ*^2^-test, *P*<0.01).

**Figure 3:**
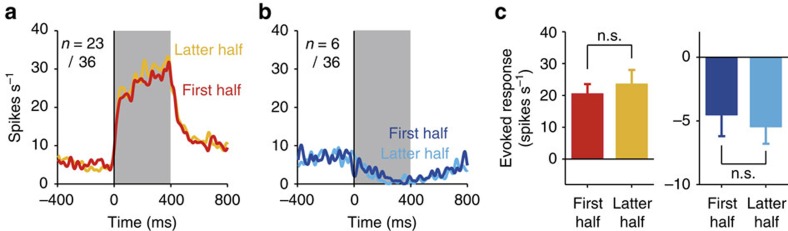
Repetition of laser light emission did not impair the firing ability of SC neurons. (**a**) Averaged activity of the 23 excited neurons in the ipsilateral condition. Spike density functions, in which each spike was replaced by a Gaussian curve (*σ*=10 ms), are aligned at the onset of the optical stimulation and shown for first half (red) and latter half trials (orange). (**b**) Averaged activity of the six suppressed neurons in the ipsilateral condition shown for first half (dark blue) and latter half trials (light blue). (**c**) Evoked-response magnitudes of the 23 excited (left) and 6 suppressed neurons (right) shown for first half and latter half trials. Error bars indicate s.e.m. n.s. indicates no significant difference (Wilcoxon signed-rank test, *P*>0.10).

We confirmed that repeated laser light stimulation did not impair the firing ability of SC neurons over the course of the experiment (Fig. 3). Figure 3a,b indicates the averaged activities of the 23 excited and 6 suppressed neurons, respectively, in the ipsilateral SC. Their activities were shown for the first half and latter half of the trials in which the laser light was emitted in the SC. In both the excited and suppressed neurons, the magnitudes of the evoked responses were not significantly different between the first half and latter half trials (Wilcoxon signed-rank test, *P*>0.10; Fig. 3c).

### Saccadic eye movement evoked by optical stimulation

**Figure 4:**
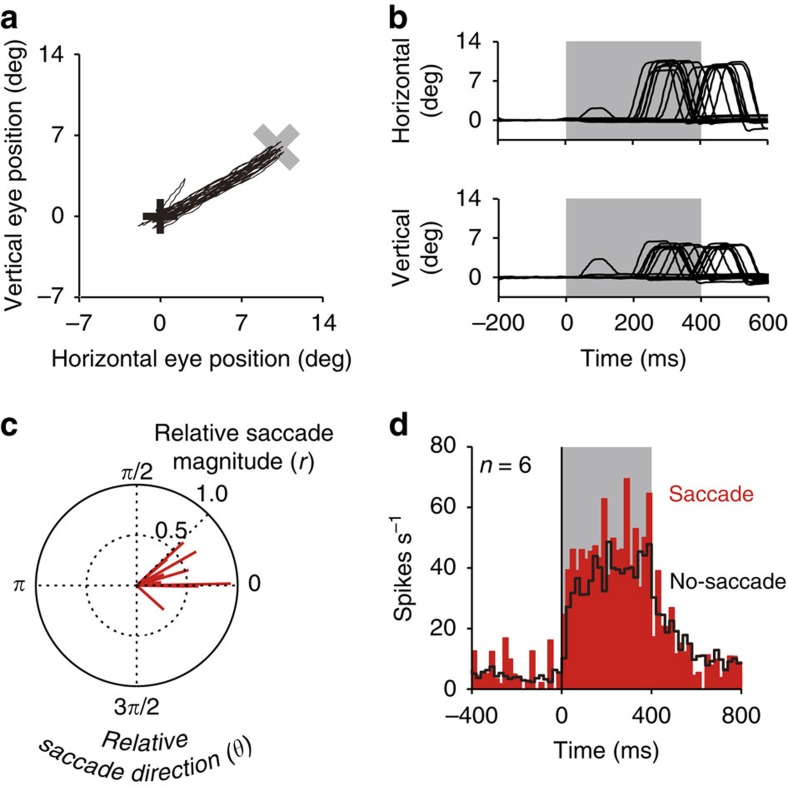
Saccadic eye movements evoked by the optical stimulation in the fixation task. (**a**) Trajectories of eye positions at an example stimulation site in the ipsilateral condition. Black and grey crosses indicate the fixation point and the centre of the RF, respectively. (**b**) Horizontal and vertical eye traces at the example stimulation site. (**c**) Polar plot of the magnitude (*r*) and direction (*θ*) of evoked saccades relative to the RF centre of stimulation sites. Red lines indicate the averaged vector of evoked saccades at each stimulation site (*n*=15). Saccade towards the RF centre is represented by (*r*, *θ*)=(1.0, 0). (**d**) Averaged activity of the six neurons in the ipsilateral SC that were excited by the optical stimulation. The averaged activity was shown for trials in which a saccadic eye movement was evoked (red) and for those in which a saccadic eye movement was not evoked (black).

The above data suggest that our optogenetic approach successfully modulated SC neuron activity in a pathway-selective manner. We next examined whether our approach was sufficient to induce behaviorual modulations. We found that optical activation of FEF axon terminals often evoked saccadic eye movements towards the response fields (RF) corresponding to the stimulation sites in the SC (see Fig. 4a,b for an example stimulation site). Evoked saccades were observed only in the ipsilateral condition (ipsilateral condition, 15 of 44 stimulation sites; contralateral condition, 0 of 42 stimulation sites; no-ChR2 condition, 0 of 13 stimulation sites), which was consistent with the expression pattern of ChR2 in the SC (Fig. 1c). The proportion of stimulation sites in which saccades were evoked was significantly higher in the ipsilateral condition than in the contralateral and no-ChR2 conditions (*χ*^2^-test, *P*<0.05).

Figure 4c shows the averaged magnitude and direction of evoked saccades relative to the RF centre of each stimulation site (*n*=15). As a population, the magnitude was significantly smaller than the eccentricity of the RF centre (Wilcoxon signed-rank test, *P*<0.01), suggesting that the power of the optical stimulation was not enough to evoke full saccades that reach the eccentricity. In addition, the direction of saccades was not exactly the same as that of the RF centre. This may be explained by two features of our optrode. First, an optic fibre was attached to a tungsten recording electrode extending 200 μm beyond the fibre end. Thus, there was a physical gap between the optically stimulated site and the recording site. Second, laser light diffuses so that a relatively wide area of the SC could be stimulated. This may also contribute to the discrepancy between the affected region and the recording site.

We next examined the relationship between the optogenetically evoked neuronal modulation and saccadic eye movements. At six stimulation sites in the ipsilateral SC where we recorded neuronal activity that was significantly excited by the optical stimulation, the stimulation sometimes evoked a saccadic eye movement but sometimes not. We found that, as a population, the recorded neurons were more strongly excited when a saccadic eye movement was successfully evoked than when it was not evoked (bootstrap test, *P*<0.05; Fig. 4d). This suggests a causal relationship between SC neuron excitation and the evoked saccadic eye movements.

### Effect of optical stimulation on saccade latency

**Figure 5:**
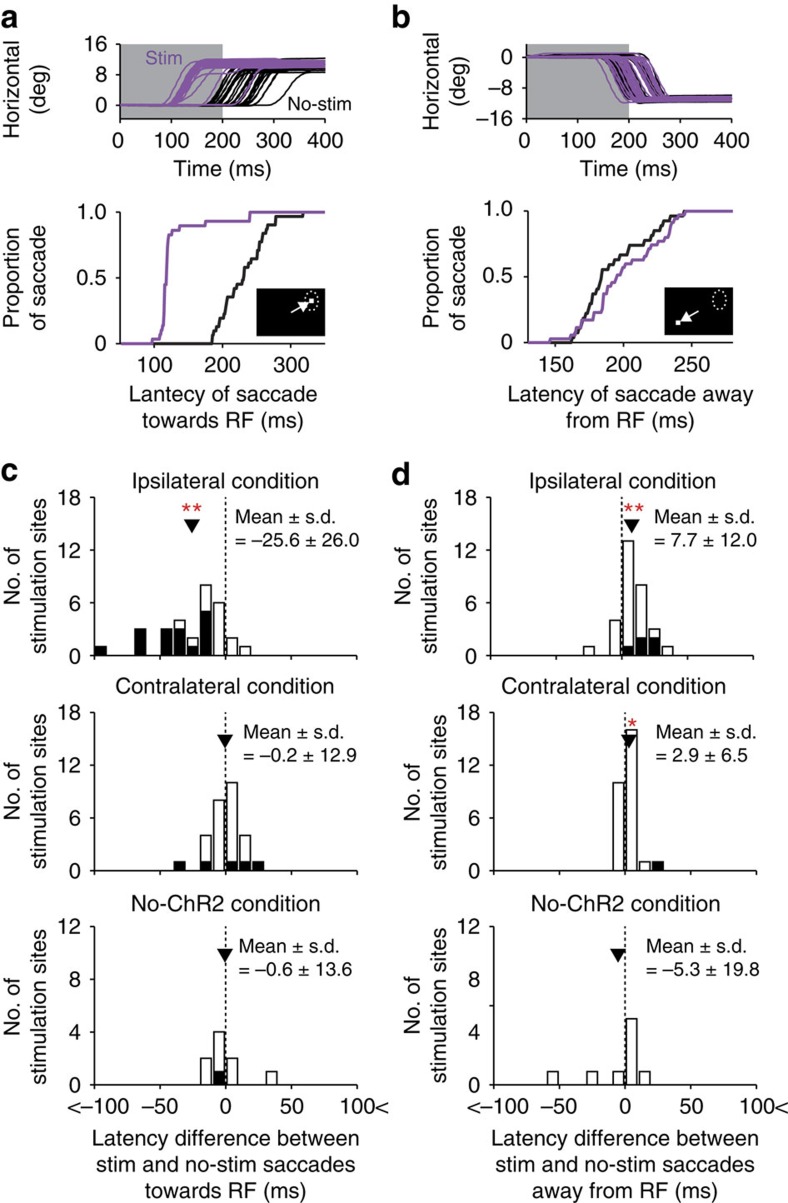
Effect of the optical stimulation on saccade latency in the visually guided saccade task. (**a**,**b**) Horizontal eye traces (top) and cumulative distribution of the latency (bottom) of saccades towards the RF (**a**) and those away from the RF (**b**) at an example stimulation site in the ipsilateral condition. Purple and black curves indicate stimulated and non-stimulated saccades, respectively. Insets indicate the configuration of the RF and saccadic target. (**c**,**d**) Distribution of the difference in saccade latency between stimulated and non-stimulated saccades towards the RF (**c**) or away from the RF (**d**) in the ipsilateral (*n*=30, top), contralateral (*n*=28, middle) and no-ChR2 conditions (*n*=9, bottom). The difference was calculated for each stimulation site. Negative values indicate shorter latencies for stimulated saccades. Filled bars indicate stimulation sites showing a significant difference between stimulated and non-stimulated saccade latencies (Wilcoxon rank-sum test, *P*<0.05). Arrowheads indicate the mean differences. Double and single asterisks indicate a significant deviation from 0 (Wilcoxon signed-rank test, *P*<0.01 and *P*<0.05, respectively).

To measure behavioural modulations more sensitively, we next optogenetically stimulated FEF-SC signalling while the monkey was performing a visually guided saccade task (Fig. 1e) and analysed the saccade latency. The optical stimulation (200 ms duration) started simultaneously with the onset of a saccadic target that was presented inside or outside the RF. The stimulation was delivered in half of the trials and was not delivered in the other half trials. In the ipsilateral condition, we performed the experiment at 30 stimulation sites in the SC. We found that stimulation of the FEF-SC pathway decreased the latency of saccades towards the RF and increased the latency of saccades away from the RF (see Fig. 5a,b for an example stimulation site). Figure 5c,d shows the distribution of the difference in saccade latency between stimulated and non-stimulated saccades towards the RF and away from the RF, respectively. The mean difference was significantly smaller than 0 for saccades towards the RF (mean±s.d.=−25.6±26.0 ms, Wilcoxon signed-rank test, *P*<0.01; Fig. 5c, top), indicating that the latency was shorter for stimulated saccades. For saccades away from the RF, on the other hand, the mean difference was significantly larger than 0 (mean±s.d.=7.7±12.0 ms, Wilcoxon signed-rank test, *P*<0.01) (Fig. 5d, top), indicating that the latency was longer for stimulated saccades. These results suggest that the pathway-selective optogenetic stimulation facilitated saccades towards the RF and suppressed those away from the RF.

The effect of the optical stimulation on saccade latency (that is, difference in saccade latency between stimulated and non-stimulated saccades) in the contralateral (28 stimulation sites) and no-ChR2 conditions (9 stimulation sites) was weaker and not significant in several cases (contralateral condition, saccades towards the RF, mean±s.d.=−0.2±12.9 ms, Wilcoxon signed-rank test, *P*>0.05, saccades away from the RF, mean±s.d.=2.9±6.5 ms, Wilcoxon signed-rank test, *P*<0.05; no-ChR2 condition, saccades towards the RF, mean±s.d.=−0.6±13.6 ms, Wilcoxon signed-rank test, *P*>0.05, saccades away from the RF, mean±s.d.=−5.3±19.8 ms, Wilcoxon signed-rank test, *P*>0.05; Fig. 5c,d, middle and bottom). The mean latency difference of saccades towards the RF was significantly smaller in the ipsilateral condition than in the contralateral and no-ChR2 conditions (Wilcoxon rank-sum test, *P*<0.01; Fig. 5c), while the mean latency difference of those away from the RF was larger in the ipsilateral condition than in the contralateral and no-ChR2 conditions (Fig. 5d), although it failed to achieve a significant level between the ipsilateral and the no-ChR2 conditions (Wilcoxon rank-sum test, ipsilateral versus contralateral, *P*<0.05, ipsilateral versus no-ChR2, *P*=0.06). Thus, the magnitude of the stimulation effect was stronger in the ipsilateral condition than in the contralateral and no-ChR2 conditions.

## Discussion

Using a pathway-selective optogenetic approach, we demonstrated that stimulation of the FEF-SC pathway, among the complex oculomotor network, is sufficient not only to modulate SC neuron activity, but also to initiate saccadic eye movements in macaque monkeys. The effect on oculomotor behaviour depended on the direction of the saccade with respect to the RF, and was facilitative for saccades towards the RF and suppressive for those away from the RF. The effects of optogenetically stimulating the FEF-SC pathway on neuronal activity were somewhat less consistent. Although many of the recorded SC neurons (23/36) were excited by the corticotectal stimulation, a smaller fraction (6/36) were suppressed. It is possible that this heterogeneity might be mediated by underling differences in cell-type-specific origin of cortical inputs^18^. It is also possible, for example, that the inhibitory effect was mediated via interneurons in the SC.

Neurons in the primate FEF are known to show several types of discharges that are related to visual stimulation and saccadic eye movements^19^,^20^. Using antidromic stimulation, some studies identified FEF neurons projecting to the SC and found that these neurons transmit visual- and saccade-related signals to the SC^21^,^22^,^23^. Although microstimulation and pharmacological manipulation of the FEF have shown its contribution to oculomotor behaviour, these techniques are not sufficient to fully understand the roles of the signals transmitted through the FEF-SC pathway because they affect all output pathways from the FEF. Using pathway-selective optogenetics, here we directly stimulated the FEF-SC pathway and found that this stimulation evoked saccadic eye movements. Our findings clearly demonstrate the causal relationship between the signals transmitted through the FEF-SC pathway and saccadic eye movements.

The latency of optogenetically evoked saccades during the fixation task was relatively longer (mean±s.d.=247.3±120.8 ms, see Fig. 4b for a representative) than that of saccades elicited by electrical microstimulation of the SC or FEF^24^,^25^. One possible explanation for the longer latency is that because the neuronal modulation evoked by the stimulation gradually increased during laser light emission (Fig. 2b), a saccadic eye movement might only occur after the activity reaches a threshold. Consistent with this idea, we found that SC neurons typically exhibited less excitation in trials in which optical stimulation failed to elicit saccadic eye movements (Fig. 4d). In these cases, the neuronal modulation might not have reached the required threshold to initiate behaviour. Another explanation for the discrepancy in latency between optogentic and direct electrical microstimulation may be that the optical stimulation of FEF axons might cause the back-propagating activation of FEF neurons, which in turn could evoke a saccadic eye movement through an indirect neural pathway such as the corticobasal ganglia circuit. Although pathway-selective optogenetics has been used in rodents, the effect of back-propagating activation is not yet fully understood. Further studies are necessary to test whether pathway-selective optogenetics elicits back-propagating neuronal modulations and, if so, how the neuronal modulations influence behaviour.

Over the decades, electrical microstimulation and pharmacological manipulation techniques have been used as tools to modulate neuronal activity in various brain regions, permitting investigators to establish causal links between neuronal activity and behaviours. These methodologies, however, cannot selectively target the activity (that is, the transmitted signal) of a particular pathway connecting two regions. The advent of pathway-selective optogenetic approaches has enabled investigators to overcome this issue in rodents and now, as we have demonstrated, in nonhuman primates. Nonhuman primates are widely utilized as animal models of various normal behaviours and neurological disorders. Thus, the present pathway-selective optogenetic approach will provide a powerful strategy for elucidating the roles of targeted neural pathways in primate behaviours.

## Methods

### Animals

Two adult rhesus monkeys (*M. mulatta*; monkey LX, male, 7 years of age, 7.0 kg; monkey GN, male, 10 years of age, 9.5 kg) were used for the present experiments. All procedures for animal care and experimentation were approved by the Institutional Animal Care and Use Committee of Primate Research Institute, Kyoto University (Permission Number: 2014-080), and complied with the Guidelines for Care and Use of Nonhuman Primates by Primate Research Institute, Kyoto University (2010).

### Surgery

A plastic head holder and two recording chambers were fixed to the skull under general anaesthesia and sterile surgical conditions. One recording chamber was placed over the midline of the parietal lobes to be aimed at the SC. The other recording chamber was placed over the frontal lobe and tilted laterally by 45 degree to be aimed at the FEF. The head holder and recording chambers were embedded in dental acrylic resin that covered the top of the skull and were connected to the skull with plastic screws.

### Behavioural tasks

Behavioural task events and data acquisition were controlled with the TEMPO system (Reflective Computing). The monkey sat in a primate chair facing a frontoparallel computer monitor in a sound-attenuated and electrically shielded room. Eye movements were monitored using an infrared eye-tracking system (Eye-Trac 6, Applied Science Laboratories) by sampling at 240 Hz.

The monkey performed a fixation task (Fig. 1d). Trials began with the appearance of a central fixation point (0.5 degree), and the animal was required to fixate the point. After 500–1,100 ms of fixation (early-fixation period), a blue laser light (400 ms duration) was shone into the SC in half of the trials and was not shone in the other half trials. Trials with and without laser light emission were randomly interleaved. The laser light emission was followed by a further fixation period (late-fixation period, 500–1,100 ms). The total duration of fixation was 2,000 ms, including the early-fixation period, laser light duration and late-fixation period. The fixation was required within a ±2.5 degree window. If the monkey correctly performed the fixation, a liquid reward was delivered. Even if the fixation was broken, the monkey obtained the reward in trials in which the optical stimulation was presented. All trials were presented with a random intertrial interval that averaged 2.5 s (2.0–3.0 s). The number of trials with the optical stimulation was 11–52 (mean±s.d.=20.1±6.2 trials) for each recording session.

The monkey also performed a visually guided saccade task (Fig. 1e). Trials began with the appearance of a central fixation point. After 1,000 ms of fixation, the fixation point disappeared, and a saccadic target was presented inside or outside the RF of a single- or multi-unit activity at the stimulation site in the SC. A blue laser light (200 ms duration) was shone into the SC simultaneously with the onset of the saccadic target in half of the trials and was not shone in the other half trials. Trials with and without laser light emission were randomly interleaved. The monkey was required to make a saccade to the target to obtain a liquid reward. The number of trials with the optical stimulation was 22–79 (mean±s.d.=51.3±12.3 trials) for each recording session. The fixation task and visually guided saccade task were run in separate blocks.

### Viral vector

The AAV2-CMV-ChR2-EYFP vector (5.0 × 10^12^ genome copies per ml) was produced by the helper-free triple transfection procedure and was purified using affinity chromatography (GE Healthcare). Viral titre was determined by quantitative PCR using Taq-Man technology (Life Technologies). The transfer plasmid (pAAV-CMV-ChR2-EYFP-WPRE) was constructed by inserting ChR2(H134R)-EYFP gene (kindly provided by Dr K. Deisseroth) and WPRE sequence into an AAV backbone plasmid (pAAV-CMV, Stratagene).

### Viral vector injection

We first identified the FEF, with the aid of magnetic resonance imaging (MRI), using standard electrophysiological techniques (see the section, ‘Electrophysiology') and the following two criteria. (1) Neurons exhibit characteristic discharges evoked by visual stimuli or saccades into a particular direction and amplitude. (2) Electrical microstimulations (<50 μA, 0.2-ms cathodal pulse, 333 Hz, 100 ms duration) evoke saccadic eye movements with a particular direction and amplitude. After the identification, the viral vector was injected into the FEF by pressure through a microsyringe (Hamilton). Three penetrations were made into the left FEF at least 1.5 mm apart from each other. For each penetration, 1.0–1.5 μl of the vector was introduced at two or three different depths with a rate of 0.1 μl min^−1^.

### Laser light stimulation

For laser light stimulation and electrophysiological recording, we used optrodes (Doric Lenses); an optic fibre (200 μm diameter) attached to a tungsten recording electrode (0.5–2.0 MΩ impedance, 125 μm diameter) extending 200 μm beyond the fibre end. The optrode was advanced towards the SC by an oil-driven micromanipulator (MO-97-S, Narishige) through a stainless-steel guide tube, which was inserted into the brain via the dura. The SC was identified, with the aid of MRI, based on the characteristic neuronal activity recorded through the attached electrode (that is, neuronal discharges evoked by visual stimuli or saccades into a particular direction and amplitude) and based on evoked saccadic eye movements with a particular direction and amplitude by electrical microstimulations (<50 μA, 0.2 ms cathodal pulse, 333 Hz, 100 ms duration). Using a laser light source (COME2, Lucir), we shone a blue laser light (473 nm wavelength, <1,100 mW mm^−2^) into the SC. The light intensity was within the range of the intensity that was used in a recent study of Cavanaugh *et al*.^7^ who successfully induced a behavioural modulation by a non-pathway-selective optogenetic approach in nonhuman primates^7^. We confirmed that the laser light with this intensity did not affect the activity of SC neurons (Fig. 2c–e) nor did impair their firing ability (Fig. 3).

### Electrophysiology

Electrophysiological recordings were performed using the optrodes. Extracellular potentials were amplified and band-pass-filtered (100 Hz to 8 kHz) using a multichannel processor (MCP Plus 8, Alpha Omega) and were isolated online using a voltage–time window discrimination system (ASD, Alpha Omega). The time of occurrence of each action potential was stored with 1 ms resolution.

We recorded single-unit activity from 36 neurons in the ipsilateral SC, 36 neurons in the contralateral SC and 9 neurons in the no-ChR2 condition. These neurons were distributed primarily in the intermediate layer of the SC where ChR2 was densely expressed (Fig. 1c). The eccentricity of their RF positions ranged from 4.1 to 26.9 degree (mean±s.d.=12.3±6.8 degree) in the ipsilateral condition, from 6.2 to 21.2 degree (mean±s.d.=12.8±4.3 degree) in the contralateral condition, and from 2.5 to 13.3 degree (mean±s.d.=6.0±4.0 degree) in the no-ChR2 condition. The mean eccentricity was not significantly different between the ipsilateral and contralateral conditions (Wilcoxon rank-sum test, *P*>0.10) but was significantly different between the ipsilateral and no-ChR2 conditions (Wilcoxon rank-sum test, *P*<0.01).

Extracellular axonal action potential typically has a shorter duration than somatic action potential and tends to be triphasic^26^. We avoided to record single-unit activity with such an action potential.

### Data analysis

We defined the response of SC neurons evoked by the optogenetic stimulation as the discharge rate during laser light emission minus the discharge rate during 200–0 ms before the onset of the laser light. The statistical significance of the response was tested using Wilcoxon signed-rank test based on the response magnitudes in individual trials.

We conducted a bootstrap test to assess the statistical significance of the difference in optogenetically induced neuronal excitation between trials in which a saccadic eye movement was successfully evoked (saccade-evoked trials) and those in which a saccadic eye movement was not evoked (no-saccade-evoked trials; Fig. 4d). For each of the six neurons that were excited by the optical stimulation, the trials were randomly resampled with replacements to form a new bootstrap data set, which had the same number of trials as the original data set. The bootstrap data sets of the six neurons were combined, and the averaged magnitude of their excitation induced by the optogenetic stimulation was compared between the saccade-evoked and the no-saccade-evoked trials. Such random resampling and comparison were repeated 1,000 times. If the averaged magnitude was larger or smaller in the saccade-evoked trials than in the no-saccade-evoked trials in >975 repetitions, the magnitude difference was regarded as significant (*P*<0.05).

### Histology

Monkey LX was deeply anaesthetized with pentobarbital sodium (50 mg kg^−1^, intravenously) and perfused transcardially with 0.1 M PBS, followed by 10% formalin in PBS. The brain was removed from the skull, blocked into pieces and equilibrated with 30% sucrose in PBS. Frozen sections were cut at 50 μm thickness in the coronal plane. To visualize the immunoreactive signals of ChR2-EYFP and NeuN, the sections were immersed in 1% skim milk for 1 h and incubated overnight with rabbit anti-green fluorescent protein antibody (1:1,000 dilution, Life Technologies) and mouse monoclonal anti-NeuN antibody (1:1,000 dilution, Millipore) in PBS containing 1% normal donkey serum. The sections were then incubated in the same fresh medium containing Alexa488-conjugated donkey anti-rabbit IgG (1:400 dilution, Jackson ImmunoResearch) and Cy3-conjugated donkey anti-mouse IgG (1:400 dilution, Jackson ImmunoResearch). Images of the sections were digitally captured using an optical microscope equipped with a high-grade charge-coupled device camera (Biorevo, Keyence).

## Additional information

**How to cite this article:** Inoue, K. *et al*. Neuronal and behavioural modulations by pathway-selective optogenetic stimulation of the primate oculomotor system. *Nat. Commun.* 6:8378 doi: 10.1038/ncomms9378 (2015).

## Supplementary Material

Supplementary Information Supplementary Figure 1
