# Supplementary material for: Neuronal and behavioural modulations by pathway-selective optogenetic stimulation of the primate oculomotor system
Source: Nat Commun. 2015 Sep 21;6:8378. doi: 10.1038/ncomms9378 (PMC4595751; doi:10.1038/ncomms9378)
Supplement: Supplementary Information — Supplementary Figure 1 [file ncomms9378-s1.pdf]

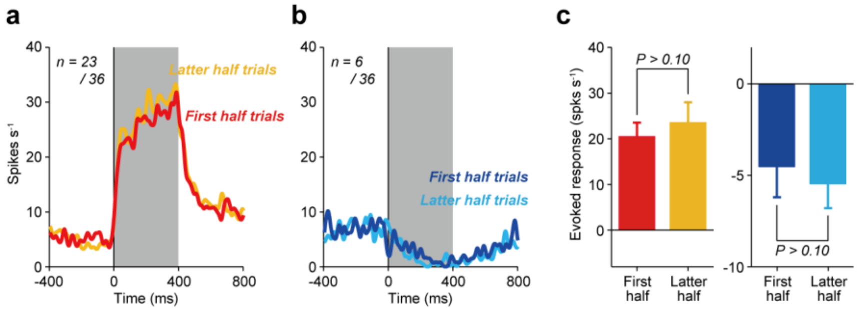

Supplementary Figure 1

Repetition of laser light emission did not impair the firing ability of SC neurons. (**a**) Averaged activity of 23 neurons displaying a significant excitation evoked by optical stimulation in the ipsilateral condition. The activity is shown for first half trials and latter half trials. (**b**) Averaged activity of 6 neurons displaying a significant inhibition evoked by the stimulation. (**c**) In both the 23 excited neurons (left) and the 6 inhibited neurons (right), the magnitudes of the evoked responses were not significantly different in the first and latter half trials (Wilcoxon signed-rank test, $P > 0.10$). This suggests that the firing ability of these neurons was not impaired by the repetition of laser light emission.
